# Launching Stealth AutoGuide^TM^ robot for stereotactic biopsy procedures in a neurosurgical centre: learning curve and workflow optimization

**DOI:** 10.3389/frobt.2024.1437568

**Published:** 2024-12-17

**Authors:** Marcus Barth, Etienne Holl, Fabian Flaschka, Sila Karakaya, Vitus Körbler, Melanie Pichlsberger, Stefan Wolfsberger, Alexander Micko

**Affiliations:** Department of Neurosurgery, Medical University of Graz, Graz, Styria, Austria

**Keywords:** autoguide, stereotactic biopsy, learning curve, workflow, prone position, accuracy

## Abstract

**Background:**

Accurate histological analysis is crucial for confirming intracerebral neoplasia due to the diverse array of potential diagnoses presented by imaging. In the realm of biopsy techniques, the use of robot-based systems is on the rise, primarily owing to their heightened targeting accuracy. The objective of this study was to elucidate the practicality, learning curve and workflow associated with robot-guided biopsies upon their introduction to a neurosurgical centre.

**Materials and methods:**

In March 2022, the neurosurgical department at our institution adopted the Medtronic Stealth AutoGuide™ cranial robotic guidance platform, a miniaturized robotic guidance device designed for stereotactic procedures. Four experienced neurosurgeons underwent training with the Stealth AutoGuide™ system, after which 51 consecutive biopsies were performed. The evaluation encompassed entry and target accuracy, preoperative setup time, time required for the biopsy procedure, and overall operating time. Statistical analysis was conducted to identify any differences between the initial 26 and subsequent sets of 25 patients, with the aim of identifying changes in workflow.

**Results:**

The study included all patients necessitating a diagnostic biopsy for intracerebral tumours, with only one patient excluded due to the inaccessibility of the intended target point caused by tumour calcification. Notably, there were no significant differences between the first 25 and last 26 patients in the median time from incision to the first biopsy (overall: 11.5 min, IQR 9.03–15.0), the procedure time (overall: 30.0 min, IQR 23.5–46.5), median accuracy at entry (overall: 2.05 mm, IQR 0.8–3.8), or target point (overall: 2.2 mm (IQR 1.6–3.7). However, a significant reduction in robot setup time was observed between the two groups, median 2.69 min versus 1.17 min, respectively (p ≤ 0.001).

**Conclusion:**

The deployment of the robotic biopsy system, Stealth AutoGuide™, showed high feasibility, steep learning curve due to uncomplicated technical handling using our standardized operative workflow. Therefore, even in prone position a high diagnostic yield was achieved. Overall, the Stealth AutoGuide™ system facilitated biopsies in traditionally challenging regions with concise procedure time and surgeon-independent high accuracy.

## Introduction

Advancements in medical technology have profoundly reshaped the landscape of neurosurgery, especially in the sphere of stereotactic intracranial biopsies. The confirmation of intracerebral tumours requires a precise histological analysis owing to the vast array of potential diagnoses that are derived from imaging. On the basis of pathologist’s diagnosis of these tissue samples, oncologists can tailor patient-specific targeted therapies, representing a cornerstone of contemporary cancer treatment practices.

One crucial consideration lies in the imperative requirement for precision to ensure the efficacy of these procedures. However, many biopsy needle procedures for obtaining tissue specimens are currently performed with limited accuracy, i.e., by using a mechanical arm. Such methods hold the potential for procedural setbacks, ranging from inconclusive tissue sampling to severe complications like cerebral haemorrhage resulting in subsequent neurological deficits. In context of stereotactic needle biopsies, inaccuracy is associated with the acquisition of non-diagnostic samples in up to 24% of stereotactic biopsy series (Dammers et al., 2010; Dammers et al., 2008; Zoeller et al., 2009) or non-representative tumour specimen in up to 64% of cases (Aker et al., 2005; Jackson et al., 2001; Muragaki et al., 2008). To address this limitation, serial biopsies may be conducted, albeit associated with increased risk of intracranial haemorrhages (Dickerman et al., 2005). These haemorrhages have been reported in 0.3%–59.8% of cases (Dammers et al., 2008; Field et al., 2001; Grossman et al., 2005) significantly contributing to the reported morbidity rates ranging from 0% to 16.1% associated with this procedure (Dammers et al., 2010; Dorward et al., 2002; Lunsford et al., 2008; Tilgner et al., 2005).

The advent of robotic assistance systems has opened up novel pathways for precision and efficiency in neurosurgical procedures (Kaushik et al., 2020). Among the diverse techniques of performing intracranial biopsies that have demonstrated feasibility, robot-based frameless solutions have emerged as a predominant trend recently (Minchev et al., 2020; Deboeuf et al., 2023; Dlaka et al., 2021; Wang et al., 2022; Wu et al., 2021).

A notable innovation in this realm is the Medtronic Stealth AutoGuide™ cranial robotic guidance platform (Medtronic, Minneapolis, MN, United States) which has emerged as a promising tool for guiding frameless biopsies with high accuracy and straightforward handling. The Stealth AutoGuide™ system is a modular guidance system for surgical invasive tools, offering precise trajectory alignment based on predefined navigation data. It consists of two independent guidance modules, each capable of moving in two dimensions, mounted on top of one another to enable precise positioning of the biopsy needle. This configuration allows for any trajectory within a 40 × 40 mm range and up to 30° of angulation, achieving an internal precision of less than 0.1 mm. The robot is designed to automatically acquire and stabilize the desired trajectory, enabling the surgeon to operate hands-free without the need for manual adjustments. The main advantages over competing surgical systems, such as the ROSA One® Brain system (Zimmer Biomet, Jacksonville, FL, United States) and the Renishaw Neuromate® stereotactic robot (Renishaw plc, Wotton-under-Edge, United Kingdom), include its miniaturized design, ease of use, and comparatively lower cost. For further technical details, see Minchev et al., 2021 (Minchev et al., 2021).

In this retrospective study, we aim to evaluate the learning curve and workflow associated with the implementation of the Medtronic Stealth AutoGuide™ in a neurosurgical centre over a 20-month period spanning from 2022 to 2024. Robotic assistance provides the potential to transform stereotactic biopsy procedures, potentially leading to reduced procedure times and opportunities for standardization.

## Methods

### Study design

This retrospective, single centre study employed a one-armed design to analyse data spanning 20 months following the introduction of the Medtronic Stealth AutoGuide™ from 2022 to 2024. The study aimed to evaluate the learning curve and workflow associated with robot-guided biopsies upon their introduction to a tertiary neurosurgical centre. Eligible participants included all adults aged 18 or older, who underwent a stereotactic frameless biopsy using the Stealth AutoGuide™ robot device. Indications for biopsies were determined by board certified neurosurgeons following decisions made in interdisciplinary tumour board meetings. No patients were excluded following the analysis of the database. This study received approval from the institutional ethics committee (IRB number: 36-236-23/24) and was done in accordance with the principles of the Declaration of Helsinki. Informed consent was obtained from all participants.

### Data acquisition

Data acquisition was conducted within a fixed protocol for consecutive cases: Preoperatively, patients underwent cCT (SOMATOM® Force, Siemens Healthineers; non tilted axial scan, soft tissue window, slice thickness 1 mm) and cMRI scans (SIEMENS MAGNETOM 3.0T XT Numaris, Siemens Healthineers; T1 with gadolinium contrast enhancement, slice thickness 1 mm, TR 20.0 ms, TE 4.92 ms, flip angle 25°, voxel size 1 ×1 × 1 mm) with isovoxel sequences, suitable for neuronavigation (Mert et al., 2015). Utilizing the T1-weighted, gadolinium-enhanced MRI scan, stereotactic trajectories were generated using the Medtronic StealthStation™ S8 surgical navigation system (Medtronic, Minneapolis, MN, United States) based on manual plans (Marcus et al., 2020). Parameters such as bone thickness, distance from dura to target, and entry angle were measured on preoperative imaging. In the operating theatre, the patients’ skull was fixated using a Mayfield® Cranial Stabilization System (INTEGRA LifeSciences, Princeton, NJ, United States). Subsequently, optical navigation was employed, and the Medtronic Stealth AutoGuide™ was attached to the Mayfield® skull clamp. The robot required positioning within a 40 × 40 mm area and maximum angulation of ±30° to approach the trajectory (Minchev et al., 2021). The setup time (duration from handover of the robot from the OR assistant to the surgeon until the start of skin disinfection and covering), biopsy time (duration from skin incision to the first biopsy) and procedure time (duration from skin incision to complete closure) were recorded. In cases where glioma or lymphoma have been suspected, the biopsy specimen was checked for 5-ALA positivity under 405-nm-wavelength blue light (Hadjipanayis et al., 2015; Stummer et al., 2006). Within 2 h postoperatively, patients underwent CT scan as recommended (Riche et al., 2022), to anticipate procedure-related haemorrhage before transferring the patient back to ward. To assess accuracy of entry and target points as well as deviation of trajectory angle, these CT sequences were merged with the preoperative neuronavigation plan. The entry point error (EPE, distance between planned and real entry point) was measured at the centre of the burr hole, the target point error (TPE, distance between planned and real target point) was determined at the centre of the air bubble within the biopsy area. Trajectory angle error (AE) was measured as degrees of deviation to the planned trajectory.

### Patient-to-image registration

A skin-surface registration was employed in all cases according to a standard protocol (Woerdeman et al., 2007).

In prone position, we employed a strict registration workflow to minimize registration error and potential lack of diagnostic yield: In supine position, an approximation of the entry point is pre-marked using anatomical landmarks and correlations with the 3D model. Then, the patient is turned face down, the head is rotated and secured in the Mayfield® clamp so that the previously marked area becomes the highest point for visualization. For improved accessibility of the face area, the upper body is elevated without tilted neck (legs are elevated in opposite direction) so that the patient’s face is pointing slightly upwards. The camera arm is positioned on the left or right side of the operating table depending on head rotation. The navigation camera is pointed towards the patient’s face. Surface registration is conducted using the Medtronic optical pointing device marking facial aspects first and then continuing on the skull until a value of less than 1.5 mm median root mean square error (RMSE, systematic registration error of the robot) is achieved.

### Operative workflow

**FIGURE 1:**
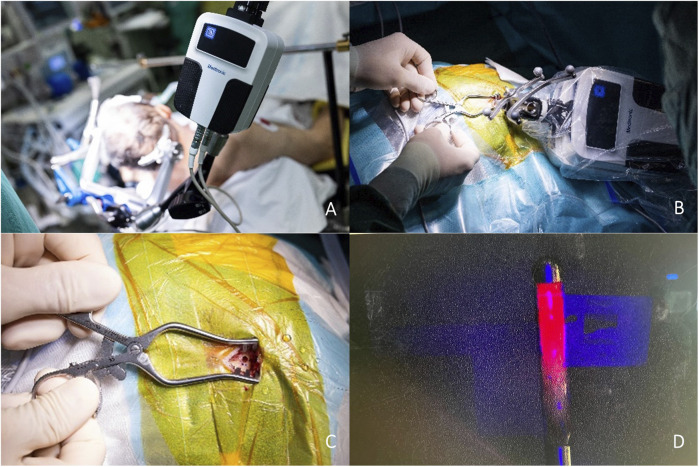
**(A)** - Robot setup, Mayfield clamp on patient head; **(B)** - Intraoperative robot positioning for biopsy; **(C)** - Burr hole using the 3.2 mm drill; **(D)** - 5-ALA fluorescence obviates the need for intraoperative histopathology decreasing procedure time.

**FIGURE 2:**
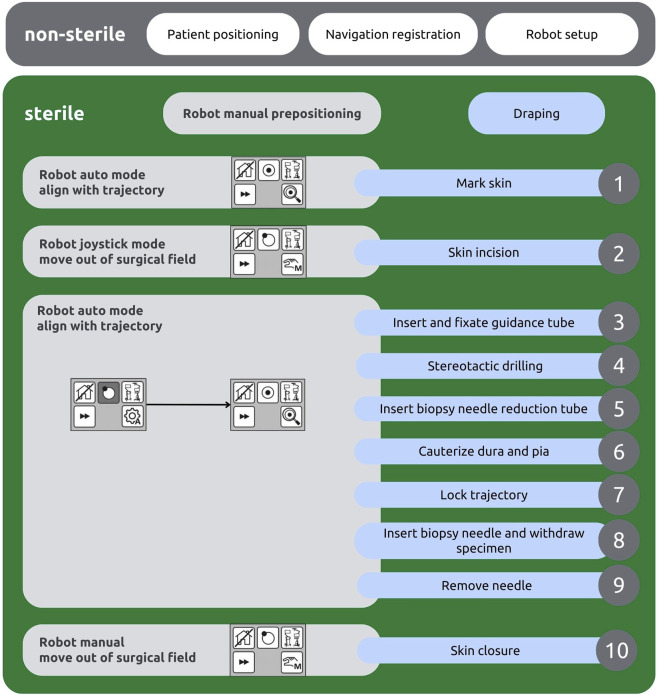
Intraoperative workflow chart depicting the robot’s tasks (left, grey) and the surgeon’s tasks (right, blue).

An ideal intraoperative workflow was developed at our department during the time of this study to optimise precision and reduce complications: Figures 1, 2

1. Starting with prepositioning of the robot in alignment with the trajectory, the entry point is marked on the skin.
2. The robot is then moved away from surgical field using manual joystick mode, so that the operating field at the entry point can be accessed. A skin incision is placed depending on the preferred burr hole width (12 mm for 3.5 mm drill, 25 mm for 7.5 mm drill) with subsequent cauterization and display of the bone surface.
3. The robot is automatically repositioned acquiring trajectory and target alignment of ≤0.1 mm and the guidance tube is then inserted and fixed to the bone.
4. A burr hole is placed by drilling with pre-set depth according to the bone thickness as measured on preoperative CT scans. Advancing in 1 mm steps reduces the risk of unwillingly opening the dura and is conducted until elastic resistance is palpated with the drill.
5. The biopsy-needle reduction tube is inserted.
6. Dura and pia are cauterized and pierced using a neuroendoscopic bipolar (Aesculap® Minop® Bipolar Fork Electrode 2.1 mm).
7. The trajectory is locked to navigate the depth advancement of the biopsy needle.
8. The biopsy needle is inserted and up to four specimen samples are withdrawn from the quadrants of the target.
9. Finally, the needle is removed.
10. Skin closure is performed as usual.

### 5-ALA, frozen section, histopathology

5-ALA has been shown to be highly sensitive for detection of suspected lymphoma and glioblastoma (Kiesel et al., 2018). Therefore, an intraoperative frozen section sample was not performed in 33 of 51 cases (64.7%) where the specimen showed high 5-ALA positivity under 405 nm light examined using the Zeiss Kinevo® 900 microscope (Carl Zeiss AG, Oberkochen, Germany). However, for other pathologies and at the surgeon’s preference, additional frozen section analysis was conducted. This supplementary step extended the procedure time, albeit ensuring comprehensive diagnostic evaluation.

### Trained surgeons

In total, four experienced neurosurgeons underwent training with the handling of the Stealth AutoGuide™. Throughout the entire investigation period, neither the setup nor the procedure underwent alterations. Preliminary differences were only apparent in the size of the applied burr hole, as the used drill provided either a 3.5 mm or a 7.5 mm tip diameter, based on surgeon preference.

### Statistical analysis

Categorical data are depicted as numbers and relative frequencies, while nonparametric data are presented as median and interquartile range (IQR). Contingency tables underwent evaluation utilizing the Fisher exact test. Group comparisons of independent samples were conducted using the non-parametric Mann-Whitney-U test. Additionally, the Kruskal-Wallis H test was employed to discern differences among the four involved neurosurgeons. A two-sided p-value <0.05 was considered statistically significant. For statistical analyses, SPSS® version 29.0 software (IBM Corp., Armonk, NY, United States) was used.

## Results

### Patients’ characteristics

The application of the Stealth AutoGuide™ robot was feasibly in all 51 consecutive patients. Histology was conclusive in all cases. Within the study cohort, Glioblastoma multiforme (WHO Grade IV) (31/51, 60.8%) was predominant, followed by Astrocytoma (Pilocytic Astrocytoma WHO Grade I, Diffuse Astrocytoma WHO Grade II, Anaplastic Astrocytoma WHO Grade III) (5/51, 9.8%), Lymphoma (Diffuse large B-cell lymphoma) (9/51, 17.6%) and Gliosis (2/51, 7.8%). Median lesion volume was 15.2 cm³ (IQR 5.55–29.25 cm³). Most patients underwent surgery in supine position (40/51, 78.4%), median head rotation from sagittal plane was 7.5° (IQR 0.0°–37.5°) and the mainly utilised drill width was 3.2 mm in 70.6% of the cases. In patients that received 5-ALA (38/51, 74.5%) preoperatively, specimen showed a strong fluorescence in 68.4%. A frozen section was performed in 35.3% of the cases. Table 1.

**TABLE 1:** Patient characteristics.

		Total frequency	IQR/Proportion (%)		Group 1	Group 2
N		51			26	25
Age (years; median; IQR)		62	54	71	59	63
Sex	Female	19	37.3		9	10
	Male	32	62.7		17	15
Histology	GBM	31	60.8		17	14
	Astrocytoma	5	9.8		2	3
	Lymphoma	9	17.6		5	4
	Gliosis	2	3.9		1	1
	Other	4	7.8		1	3
Tumour volume (cm³; median; IQR)		15.2	5.55	29.25	17.75	14.3
Position	Supine	40	78.4		19	21
	Prone	10	19.6		6	4
	Side	1	2.0		1	0
Head rotation (°; median; IQR)		7.5	0.0	37.5	20	0
Drill width	3.2 mm	36	70.6		20	16
	7.5 mm	13	25.5		4	9
5-ALA Positivity	−	5	9.8		1	4
	+	7	13.7		4	3
	++	26	51.0		17	9
Frozen section	yes	18	35.3		7	11
	no	33	64.7		19	14

(p > 0.005 between groups for “Age,” “Head rotation” and “volume”; °…degree; IQR … interquartile range).

### Accuracy and patient positioning

Evaluation of preoperative imaging revealed a median bone thickness of 7.5 mm (IQR 6.0–9.0 mm), median distance from dura to target of 36.6 mm (IQR 30.9–51.0 mm) and an angulation of the trajectory from the perpendicular axis of 8.5° (IQR 3.1°–16.0°). The median RMSE as systematic deviation provided by Medtronic StealthStation™ S8 after registration, was 1.25 mm (IQR 1.1–1.5 mm). Overall, accuracy measurements yielded an entry point error (EPE) of 2.05 mm (IQR 0.8–3.8 mm), a target point error (TPE) of 2.2 mm (IQR 1.6–3.5 mm) and a deviation from the axis of the planned trajectory of 3.0° (IQR 1.0°–5.0°) (Table 2).

**TABLE 2:** Accuracy and workflow, differences between groups.

	Median	IQR		Group 1	Group 2	P-Value
RSME [mm]	1.25	1.1	1.5	1.35	1.20	N/S
Bone thickness [mm]	7.5	6.0	9.0	7.0	8.0	N/S
Distance dura target [mm]	36.6	30.9	51.0	37.0	36.0	N/S
Angle at entry [°]	8.5	3.1	16.0	6.0	10.0	N/S
Entry point error [mm]	2.05	0.8	3.8	2.1	2.0	N/S
Target point error [mm]	2.2	1.6	3.5	2.65	1.9	N/S
Angle at entry error [°]	3.0	1.0	5.0	3.0	3.0	N/S
Setup time [min]	1.5	1.08	2.69	2.69	1.17	<0.001
Biopsy time [min]	11.5	9.03	15.0	11.21	11.5	N/S
Procedure time [min]	30.0	23.5	46.5	30.0	31.0	N/S

(°…degree; N/S … non significant; IQR … interquartile range).

**FIGURE 3:**
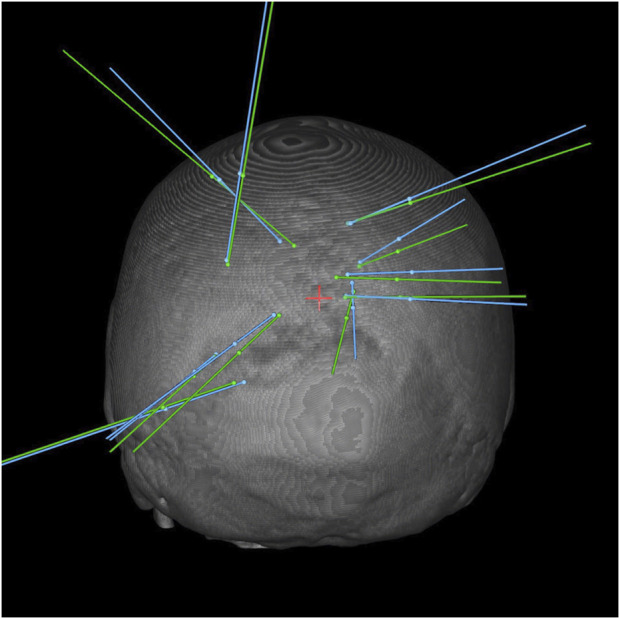
3D Bone reconstruction showing the planned (blue) and real (green) trajectories for biopsies in prone position.

Ten patients were placed in prone position, as targets in these cases were located in the parietal, occipital lobes or within the posterior fossa to minimize entry angle and distance from dura to target. In comparison with patients in supine position, RSME (1.2 mm vs. 1.3 mm, p = 1.0) showed no difference between positioning, opposing recent research (Czabanka and Misch, 2016) where surface registration could not be performed with the same accuracy in prone position. However, the angle at entry error (3.0° vs. 1.0°, p = 0.019) was found significantly lower in patients in prone position. Both, accuracy and workflow parameters were not significantly different between positionings, with entry and target point error as well as setup time being lower in prone position, and biopsy time being lower in supine positioned patients (Table 3; Figure 3).

**TABLE 3:** Comparison of supine and prone position.

	Supine (n = 40)			Prone (n = 10)			P-Value
	Median	IQR		Median	IQR		
RSME [mm]	1.3	1.1	1.45	1.2	1.1	1.5	N/S
Bone thickness [mm]	8.0	6.0	9.0	7.0	7.0	8.4	N/S
Distance dura target [mm]	36.3	30.9	48.4	35.2	32.2	54.0	N/S
Angle at entry [°]	10.0	4.5	16.5	5.5	3.1	8.0	N/S
Entry point error [mm]	2.0	0.8	3.9	1.85	0.6	3.1	N/S
Target point error [mm]	2.25	1.6	3.5	1.95	1.2	3.7	N/S
Angle at entry error [°]	3.0	2.0	6.2	1.0	0.9	3.0	0.019
Setup time [min]	1.5	1.21	2.69	1.05	0.66	2.75	N/S
Biopsy time [min]	11.5	9.25	15.0	12.95	9.03	14.58	N/S
Procedure time [min]	30.5	22.0	51.0	27.57	25.0	44.0	N/S

(°… degree; N/S…non significant; IQR interquartile range).

### Workflow and learning curve

**FIGURE 4:**
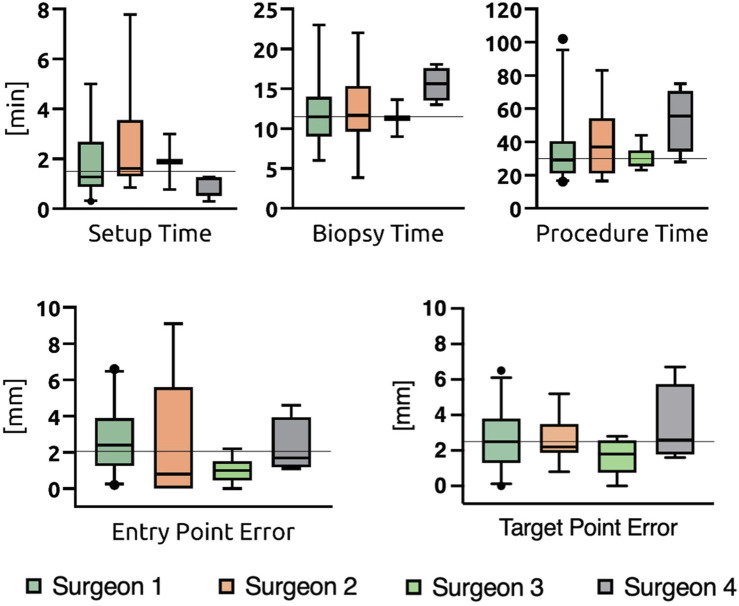
Differences in accuracy between surgeons.

Workflow measurements revealed a median setup time of 1.5 min (IQR 1.08–2.69 min), median biopsy time was 11.5 min (IQR 9.03–15.0 min) and median procedure time was 30.0 min (IQR 23.5–46.5 min). For further analysis patients were divided into two groups depended on the date of surgery. Group 1 being the first half of the patient cohort (n = 26) and Group 2 the second half (n = 25). The groups showed a congruent matching of baseline characteristics (Table 1). Regarding accuracy, entry and target point error non-significantly decreased over time (EPE 2.1 mm–2.0 mm, p = 0.472; TPE 2.65 mm–1.9 mm, p = 0.453). Analysis of the workflow revealed a significant decline in setup time (2.69 min–1.17 min, p < 0.001). However, no significant changes in biopsy and procedure time have been found. For both, accuracy and workflow analysis, no significant differences between the four trained surgeons could be found. (Figure 4; Table 2).

### Complications

Intraoperative bleeding occurred in one case (2%) during tumour sampling where the target was located close to the pineal gland and histopathological analysis revealed a pineal parenchymal tumour of intermediate differentiation. Postoperative cranial CT scan showed no sign of mass effect, but intraventricular blood accumulation. Therefore, an external ventricular drainage was placed, and the patient was transferred to ICU for 1 day. The patient could be released without clinical or neurological deficit on postop day 21, without the need for a ventriculoperitoneal shunt.

## Discussion

Evaluating the learning curve and understanding workflow dynamics of this technology is paramount for optimizing patient outcomes and integrating robotic systems seamlessly into neurosurgical practice. We examined the initial 51 stereotactic biopsy cases conducted at an institutional neurosurgical department utilizing the Medtronic Stealth AutoGuide™ system, with a focus on evaluating workflow efficiency and the learning curve effect. A comparative analysis between a sample of 26 primarily treated patients and 25 consecutively treated patients revealed a significant improvement in the time required to set up the robot. No significant differences were observed either among the treating surgeons or between supine and prone positioning.

### Accuracy

Key outcome criteria regarding accuracy slightly exceeded previously reported values (Minchev et al., 2019), with a median entry point error of 2.05 mm (IQR 0.8–3.8 mm) and a median target point error of 2.2 mm (IQR 1.6–3.5 mm) across the entire sample of 51 patients. A predominant contributing factor to these results has been observed by a RMSE of 1.25 mm due to the routine use of surface registration. Other studies employed screw-based registration (Minchev et al., 2017) which we deemed less favourable due to the invasive nature of this procedure. Nevertheless, we would recommend using screws for improved registration accuracy for small lesions (volume <1 cm³) in highly eloquent areas (such as the brainstem) which were not cases in study.

Our method to measure accuracy relied on entrapped air detection on postoperative CT scans approximately 2 h postoperative to measure entry and target deviation, which might have contributed to higher entry and target point error. Alternative imaging methods such as postoperative MRI scans were not adopted at our department due to the cost-benefit trade off. Nonetheless, the median accuracy remained within an acceptable range (Zrinzo, 2012).

### Learning curve

Assessing the learning curve, we compared the first half (26 cases, Group 1) to the later biopsy cases (25 cases, Group 2). A trend toward slight improvement in accuracy measurements was noted, particularly in case of the target point error which decreased from 2.65 mm to 1.9 mm (p = 0.453), achieving a decline below the desirable threshold of less than 2 mm for stereotactic interventions. In all cases, a conclusive histologic diagnosis was obtained, reaffirming the technique’s feasibility as demonstrated in prior research (Minchev et al., 2017). Studies conducted during the development of the Stealth AutoGuide™ robot (Minchev et al., 2021) involved only a few pre-trained neurosurgeons and primarily utilized the prototype iSYS1 robot under technical supervision. In contrast, our investigation followed a more field research-oriented approach, examining the use of the Stealth AutoGuide™ system under routine clinical application. Hence, we posit that the data elucidated in this study more accurately reflect the authentic outcomes when newly integrating such robotic systems into a neurosurgical department.

### Positioning

The analysis of challenging stereotactic biopsies performed in prone position did not reveal significant deviants from supine position. Several authors observed that “Neuronavigation system precision is more accurate in supine position compared to prone position due to improved visibility of face and convexity surface for the navigation camera” (Czabanka and Misch, 2016) and “[…] stereotactic procedures should be performed with the patient in the identical position during imaging and intervention.” (Rohlfing et al., 2003). We indeed could show that process standardisation as well as the application of a user-independent robotic device can minimize difficulties of prone positioning and path the way for performing biopsies of intricate target locations.

### Workflow

Workflow measurements demonstrated a significant reduction in setup time, from 2.7 min to 1.2 min (p < 0.001), indicating enhanced efficiency in placing the robot on the Mayfield® clamp and positioning its workspace within reach for the entry point. Additionally, the individual improvement in initial robot positioning may have contributed to the slight trend of increased accuracy in Group 2. Recent RCTs underline that frameless procedures are superior to frame-based ones regarding the duration while they report procedure times of 79.1 min (Georgiopoulos et al., 2018), 59.0 min (Bradac et al., 2017) and 42.0 min (Gempt et al., 2012), respectively. Meanwhile, our biopsy and procedure times remained constant at 11.5 min and 30.0 min, respectively, suggesting these durations are among the shortest achievable with the applied technique. As frozen section analysis was conducted in 18 cases included in the analysis of procedure time, transportation, processing, and analysis of the specimen were probably prolonging factors independent of the actual biopsy procedure.

Comparative analysis of the four participating surgeons supported the concept that a robot-based biopsy technique is easily standardized and implemented across different users regardless of their level of training. No statistically significant differences were observed regarding the individual surgeons’ performance concerning outcome parameters. A prospective study comparing two different biopsy techniques, including 526 biopsies conducted over 12 years, revealed a cutoff within the learning curve concerning operating time. On average, the 12 participating surgeons required 10 cases for robot-based procedures with the Zimmer Biomet ROSA ONE® and four cases for procedures using the stereotactic mechanical arm Brainlab VarioGuide® to reduce the procedure time by approximately 15 min (Mallereau et al., 2022). The primary distinction from our technique is the minimalistic composition of the Stealth AutoGuide™ robot, which might explain its simpler adoption and the absence of inter- and intra-individual differences in biopsy and procedure time. However, future follow-up studies may identify factors contributing to improved results, given the considerable variance in sample sizes among the four surgeons.

### Limitations

The present study is subject to limitations arising from its retrospective design and the relatively modest sample size. Consequently, regression analysis could not be applied in a manner that is empirically sensible. Accuracy measurements are lacking behind due to postop confirmation of biopsy targets via CT scan instead of MR scans applied in former studies (Minchev et al., 2021).

Nonetheless, our study aimed to assess the initial impacts of implementing a new robot-based procedure and delineate the associated learning curve. However, to validate our findings, follow-up studies employing a prospective patient cohort are imperative and will provide a more comprehensive understanding of the evaluated outcomes. Expanding the pool of surgeons and incorporating users from different institutions would yield more generalizable results. This approach could help demonstrate that the device’s learning curve and effectiveness are not influenced by local factors but remain consistent across different settings and varying skill levels.

## Conclusion

The deployment of the robotic biopsy system, Stealth AutoGuide™, showed excellent feasibility, steep learning curve due to uncomplicated technical handling using our standardized operative workflow. Therefore, even in prone position a high diagnostic yield was achieved. Overall, the Stealth AutoGuide™ system facilitated biopsies in traditionally challenging regions with concise procedure time and surgeon-independent high accuracy.

## Data Availability

The raw data supporting the conclusions of this article will be made available by the authors, without undue reservation.

## Abbreviations

IRB, Institutional Review Board; TR, Repetition Time; TE, Time to Echo; 5-ALA, 5-Aminolevulinic acid; EPE, Entry Point Error; TPE, Target Point Error; AE, Angle Error; RMSE, Root Mean Square Error; IQR, Interquartile Range; ICU, Intensive Care Unit; RCT, Randomised Controlled Trial.
